# Alveolar Abscess

**Published:** 1873-07

**Authors:** Thos R. Perry


### ALVEOLAR ABSCESS.

BY THOS. R. PERRY, D. D. S.

Report of the Committee on Dental Surgery, read before the Michigan State Dental Association, for October, 1872. Read by Thos. R. Perry, D. D S.

The subject of Alveolar Abscess has been written and talked about so much, during the last few years, that your committee feel a little delicacy about making a report on it, lest they fail to interest the larger portion of this body. The object of this paper is to bring out what we know, and what we do not know about abscess. Until within a few years, it was a foregone conclusion that when a tooth had given rise to abscess it must be extracted to allow the parts to heal.

Even the author of one of our most valuable dental text books advises the extraction of a tooth, if it be the cause of abscess ; he reccommends no attempt at cure in such cases; evidently, he did not deem a cure practicable.

Of course the members of this association cannot accept such teaching at this day. Nor do we believe that the author referred to, would now advance the same views. But there they stand in the latest edition of his work. I only refer to them to show that it is but a few years since able men confessed themselves unable to cure abscess.

But few practitioners of the present day regard alveolar abscess as incurable. I presume that the majority of the members of this convention will agree that most abscesses arising from the teeth may be successfully treated, and the teeth that have caused the same rendered useful for many years. There are some who say that all can be.

Your committee do not propose to describe the various stages and symptoms of the disease, but only to give records of a few cases, in practice.

(Case No. 1.) Mrs. A., lower second bicuspid right side, abscess had been formed and lanced by the family physician twice, patient was finally advised by him to have the tooth extracted as it was of no use to her, and could not be saved. When your committee saw the case 3 years ago, the swelling had partly gone and there was a discharge through a fistulous opening, the root was thoroughly cleasned of all foreign and decayed matter, and then injected through the canal carbolic acid and cologne and repeated the treatment two or three times and in two weeks filled, all discharge had ceased and odor abated, the tooth at last accounts was doing well, about six months ago.

(Case No. 2.) Miss B. who resides in one of the New England states, called to see if anything could be done with her left superior central incisor, containing a large amalgum filling, tooth very dark with fistulous opening in gum, had been discharging more or less two years, removed filling cleansed the canal as thoroughly as possible and injected carbolic acid and iodine through canal, we continued the treatment and in about four weeks deemed it advisable to fill, three years have elapsed since the treatment, a few months since we saw the case and it was in as good condition as any of the other teeth to all appearances.

(Case No. 3.) Mr C. superior cuspid right side large cavity on distal surface, filled with gold, did notconsider the nerve exposed, although a little sensative but gave no trouble when we filled. About two and a half years after, he returned with periodentitis with slight swelling over said cuspid, it was apparent what the trouble was, and we drilled from palatine surface to nerve canal and removed the dead matter and then applied carbolic acid, we continued that treatment a few times about every other day, the tooth felt so well that the patient neglected to call as he was requested. We had reduced the inflamation almost entirely, but there was still a little secretion and offensive odor which we could not abate, the gentleman was finally obliged to leave the city on business, to be absent a month, previous to his departure we filled the root with floss and carbolic acid which remained in until we again saw him, which was about three months, he then said he was going to attend to it until it was cured ; we syringed out thoroughly, and then dried it out with bibulous paper and found the odor still there, we then pumped in chloride of zinc, saturated solution; it being a good disinfectant, for this we used a small broach wound with cotton for a piston with only a small quantity of the zinc on it, we did not do anything to it again except syringe it out with tepid water, for several days it did not seem to improve, but at the end of a week it was all well and was filled, and has remained well since, about ten months.

(Case No. 4.) Miss D. filled first superior bicuspid right side distal surface four years ago, no signs of nerve exposed at the time, in June last she came to me with severe neuralgic pains I could not locate the point of trouble, the first molar on same side had a large filling in it and, thought perhaps it might proceed from it, she applied aconite externally which seemed to relieve it for the time being, requested her if the pain did not cease to call again, she returned home and we did not see her again for two weeks, in the mean time her face had become much swollen and the bicuspid somewhat elongated, it was not difficult to decide which was the offending member, we removed the filling and found that the wall between the filling and pulp was so thin that the thermal changes produced death, after removing the decomposed matter from the canal applied carbolic acid with instructions to call next day and every day, as we were limited to one week to cure or extract, the first we could not promise, and the latter we would not do, and perhaps in the over anxiety to cure we treated too much, for at the end of the week it did not seem to be much better than when we commenced, except all inflamation had subsided and the tooth free from soreness, but that lingering odor was there with a little secretion, which did not warrant us in filling the tooth, we finally filled the root with silk and carbolic acid, and the cavity with Hills stopping and advised her to call on a good practitioner and have it treated when she arrived at her destination, we would say that neither of the last two cases had a fistulous opening, we cite these cases as they are the most troublesome we have to contend with.

Your committee could relate many more but we are occupying too much time, one case more and we are done. A young man of twenty, first inferior molar right side had been discharging through a fistulous opening for a year past, the orifice had an angry look and what would be termed in surgery a malign appearance, which led us to think that necrosis was present, the tooth had been previously filled and the roots supposed to have been at that time, we removed the filling and found one root partly filled and the other not at all, we gained free access to the roots and disinfected with carbolic acid for a few days and then injected aromatic sul. acid, and thoroughly packed the point in canal that caused the trouble with cotton, and then pressed on the piston until the acid passed through the external opening, when we withdrew the syringe and rinsed the mouth with bi-carbonate of soda, in three days after the odor had ceased, and the ulcer healing up nicely, nothing further was done except syringing with tepid water and filling the tooth with cotton, and in one week considered the tooth sufficiently well to fill permanently, which we did with very good results, even the pounding it received in filling did not produce the slightest irritation. Next day it was apparently as good as any of the teeth. Your committee think that in many cases, the over anxiety of the operator to perform a rapid cure causes him to over treat and prolongs, instead of hastens a cure, a mere letting alone sometimes will do wonders.

